# Sensitivity and specificity of immunoprecipitation of DNA containing 5-Methylcytosine

**DOI:** 10.1186/s13104-015-1069-0

**Published:** 2015-03-27

**Authors:** Cindy Y Okitsu, Chih-Lin Hsieh

**Affiliations:** Department of Urology and Department of Biochemistry, University of Southern California, 1441 Eastlake Ave., Rm 5420, Norris Cancer Center, Los Angeles, CA 90033 USA

**Keywords:** Anti- 5-methylcytosine antibody, Anti-MBD2 antibody, Immunoprecipitation of methylated DNA

## Abstract

**Background:**

Attempts to enrich or identify DNA with cytosine methylation have been commonly carried out using anti-5-methylcytosine or anti-MBD2 (methyl-CpG binding domain protein 2) antibody in immunoprecipitation (IP) assays. However, a careful and systematic control experiment to examine the sensitivity and specificity of this approach has not been reported. It is of critical importance to understand the potential pitfalls of this approach and to avoid potential misinterpretation of findings.

**Findings:**

We found that increased concentration of antibody used in the assay increased the amount of overall DNA captured as expected. The increased number of methylated cytosines in/on the DNA fragment also increased the amount of DNA captured by the antibody. Importantly, the antibody can bind to some fully unmethylated DNA fragments, even when fully methylated DNA is present in the same experiment.

**Conclusion:**

The sensitivity of anti-5-methylcytosine antibody and anti-MBD2 antibody/MBD2 binding varies with the number of methylated cytosines on the DNA target. The specificity of these antibodies can also vary for different DNA target sequences. DNA fragments with fewer CpG sites may not bind to these antibodies even when all are methylated while DNA fragments with more CpG sites may bind to the antibodies when only some of these sites are methylated. More importantly, binding of DNA to these antibodies does not always indicate the presence of DNA methylation. It is clear that false positive and false negative findings can be easily reached even though it does not nullify these convenient and simple methods completely. Great caution should be taken for the interpretation of IP results using these antibodies and rigorous confirmation by sodium bisulfite sequencing is essential.

## Findings

### Study design

The feasibility of using antibodies in immunoprecipitation (IP) to pull down DNA harboring 5-methylcytosine is evaluated in this study. DNA fragments from a plasmid with different numbers of CpG sites that can be methylated in vitro to different levels are used as targets in the IP. Plasmid DNA fragments with a single CpG methylation pattern and a mixture of plasmid DNA with different CpG methylation patterns were tested in the IP experiments. After IP, the precipitated DNA is analyzed quantitatively and qualitatively to determine the sensitivity and specificity of the assay.

## Methods

Plasmid DNA from pCLH22 was methylated *in vitro* with SssI, HhaI, and HpaII methylases individually according to the manufacturer’s instruction (NEB). The unmethylated and methylated plasmid DNA were digested with restriction enzyme MseI, followed by phenol/chloroform extraction and ethanol precipitation, and resuspended in TE (10 mM Tris pH 8.0, 1 mM EDTA) to generate DNA fragments harboring different numbers of methylated CpG sites (Table 1). IP using anti-5-methylcytosine (anti-5-methyl-C) monoclonal antibody (Diagenode) was carried out with different concentrations of the antibody and various target DNAs as described previously [1]. IP using anti-MBD2 antibody and MBD2 protein was carried out according to the manufacture’s instruction (MethylCollector kit, Active Motif). DNA pulled down by the antibody either directly (anti-5-methyl-C) or indirectly through MBD2 binding after IP was extracted for quantitation by quantitative real-time PCR (q-PCR) using six to eight TagMan probe and primer sets specific for various regions of the plasmid [2,3]. The percent pull down is calculated by dividing the DNA in the specific experiment after IP by the total chromatin fraction before the IP. A qualitative analysis by sodium bisulfite sequencing [4] of the DNA recovered after IP was also carried out to determine the methylation pattern on the DNA fragments that were precipitated by the antibody.

**Table 1:** **Target DNA fragment sizes and number of sites that can be methyalted by HhaI, HpaII, or SssI methylase**

**MseI fragment size**	**qPCR probe/primer set**	**# HhaI sites**	**# HpaII sites**	**# CpG sites**
412	LTR1	1	0	21
	LTR3	1	0	21
666	Luc1	3	4	37
185	Luc2	0	1	7
428	Luc3	1	2	30
1720	Hyg5	10	10	143
365	PBR	1	2	23

## Results

### Percent pull down increases with antibody concentration

**Figure 1:**
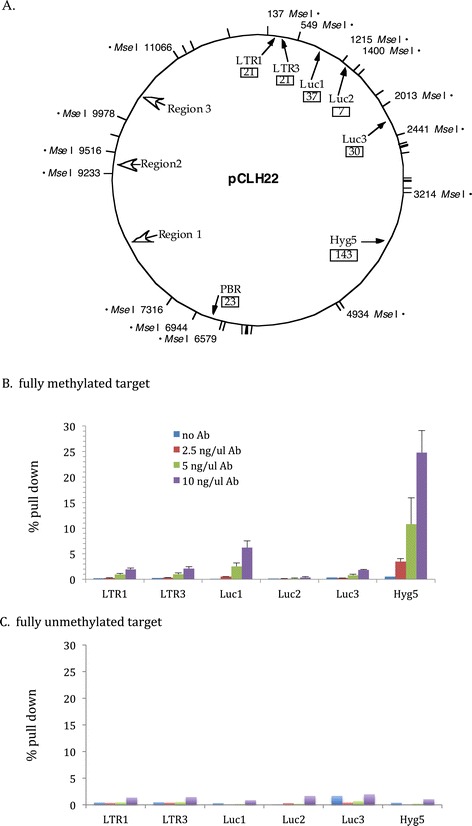
**Increase in anti-5-methyl-C antibody concentration increases the efficiency of IP.** qPCR was used to quantitate various regions of the fully methylated target DNA to determine the percentage of pull down, calculated by dividing the amount of DNA after IP by the amount of input DNA, with different concentrations of the antibody for the IP. **A)** A schematic diagram of MseI restriction sites on pCLH22. MseI restriction sites are as marked by short lines on the circle and the nucleotide positions of DNA fragments examined are as numbered. The solid arrows indicate the locations of the qPCR amplicons with the number of CpG sites in the box under the name of each probe/primer set. The hollow arrows mark the regions examined by sodium bisulfite sequencing. **B)** Percentage of IP using different concentrations of antibody and no antibody with fully methylated pCLH22 as target. **C)** Percentage of IP using different concentrations of antibody and no antibody with entirely unmethylated pCLH22 as target.

Three different concentrations of anti-5-methyl-C antibody, 2.5, 5, and 10 ng/ul, were used in each pull down experiment of 30 ul volume with 100 ng of MseI digested pCLH22 (Figure 1A) target DNA methylated at all CpG sites. Experiments using MseI digested fully unmethylated pCLH22 as target DNA were carried out as negative controls. Experiments with no antibody was done in parallel for all configurations as controls for binding of the target DNA to the protein G-sepharose beads. When 2.5 ng/ul concentration of antibody was used, from 0.3% (LTR1) to 3.5% (Hyg5) of the fully methylated pCLH22 DNA fragments were pulled down (Figure 1B). At this antibody concentration, approximately 0.1% to 0.5% of the unmethylated pCLH22 DNA fragments were pulled down from the six regions examined (Figure 1C). When 5 ng/ul concentration of antibody was used, the percent pull down was almost 1% for the LTR1 region and at 10.8% for Hyg5 region of the fully methylated pCLH22 (Figure 1B), while the unmethylated pCLH22 fragments were pulled down at the level of up to 0.7% (Figure 1C). Percent pull down increased to 1.9% for the LTR1 region and 24.8% for the Hyg5 region when antibody concentration was 10 ng/ul for fully methylated pCLH22 (Figure 1B), and was up to 2% for the unmethylated pCLH22 (Figure 1C). It is clear that the amount of DNA precipitated increases with increasing concentration of antibody for all DNA regions, except Luc2, examined for methylated DNA. While the same trend is also observed for unmemthylated DNA targets, the increases are much less notable and remain to be at what can be considered background noise level. Much more fully methylated DNA fragments with 37 and 143 CpG sites than totally unmethylated same DNA fragments (quantitated by qPCR of Luc1 and Hyg5 regions) were clearly and consistently pulled down in all the experiments. It is also noted that the DNA fragment harboring the Luc3 amplicon was pulled down by the beads along in the no antibody control at a higher rate when it is unmethylated (Figure 1C). These observation suggest that while more than seven methylated CpG sites has good potential to be distinguished from fully unmethylated DNA using the anti-5-methyl-C antibody, many more methylated CpG sites on the DNA fragment may be needed to be unequivocally identified all the time.

### Percent pull down increases with number of CpG sites in the DNA fragment

**Figure 2:**
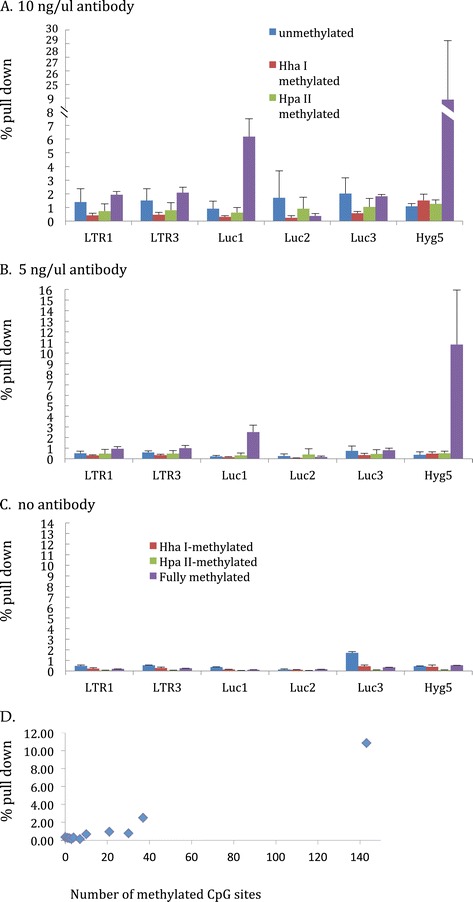
**Percent pull down by anti-5-methyl-C antibody increases with increasing number of methylated CpG sites on the target DNA fragment. A)** Average % pull down from multiple experiments using 10 ng/ul of antibody in the IP assays with differently methylated target DNA. Six regions quantitated by the TaqMan assay in five different MseI restriction fragments are presented. The number of CpG sites on each MseI restriction fragment and target DNA methylated with HhaI, HpaII, or SssI methylase harbor different numbers of methylated CpG sites as indicated in Table 1. **B)** 5 ng/ul of antibody was used in the IP assays. **C)** Parallel IP assays of **A)** and **B)** with no antibody as negative controls. In these three illustrations, the Y-axis is % pull down. **D)** Plot of methylated CpG sites on the DNA fragment (X-axis) and the % pull down (Y-axis) from the IP assays using 10 ng/ul antibody.

Different MseI digested DNA fragments from pCLH22 harbor different numbers of CpG sites (Table 1). Also, SssI, HhaI, and HpaII methylases methylate different numbers of CpG sites on the same DNA fragment based on their recognition sequences (Table 1). Three concentrations of anti-5-methyl-C antibody, 2.5, 5, and 10 ng/ul, were used in each pull down experiment of 30 ul volume with 100 ng of MseI digested pCLH22 target DNA that was methylated at all CpG sites, at only HhaI sites, and at only HpaII sites. Fully unmethylated pCLH22 as target DNA was used as a negative experimental control. Experiments with no antibody are done in parallel to determine the background due to the beads used in the assay. Nearly 25% of the fully methylated DNA fragment was detected by the Hyg5 probe/primer set as pulled down by the anti-5-methyl-C antibody at 10 ng/ul concentration (Figure 2A). This DNA fragment is 1720 bp in length and has 143 CpG sites (Table 1). In the same IP experiment, less than 0.4% of the DNA fragment was pulled down as detected by the Luc2 probe/primer set (Figure 2A), and this DNA fragment is 185 bp in length with seven CpG sites (Table 1). About 1.5% of the 1720 bp DNA fragment was pulled down by the antibody when 10 of the 143 CpG sites on the fragment were methylated by the HhaI methylase (Figure 2A), indicating that the long length of the DNA does not favor the capability of antibody binding to the fragment. While DNA fragments with 21 and 30 methylated CpG sites (assayed by LTR1, LTR2, and Luc3) were pulled down at around 2% level, the same DNA fragments were also pulled down at a similar or even higher level when they are unmethylated (Figure 2A). Similar results were found with all three concentrations of antibody (Figures. 2A, B, and data not shown). The 5-methyl-C antibody only consistently pulled down more methylated DNA than unmethylated DNA when more than 30 methylation sites are present on the DNA fragment (Figures 2A and B), reflecting the limitation of the antibody in distinguishing methylated and unmethylated DNA. Regardless of the antibody concentration used, it appears that the amount of DNA fragment pulled down by the antibody increases with increasing numbers of methylated CpG sites on the fragment (Figure 2D).

### Specificity of anti-5-methyl-C antibody is not absolute

In theory, only molecules with cytosine methylation should be pulled down by the antibody if the antibody is specific. The specificity of the anti-5-methyl-C antibody was examined by mixing differently methylated DNA for the IP. Equal amounts of fully methylated, HhaI-methylated, HpaII-methylated, and fully unmethylated pCLH22 DNA after digesting with MseI restriction enzymes were mixed for the IP using anti-5-methyl-C antibody at 5 ng/ul concentration. The methylation patterns in three different regions of the DNA were determined by the sodium bisulfite sequencing method to identify whether methylation exists at the HhaI only, HpaII only, or all CpG sites on the DNA pulled down by the antibody.

Region 1 studied by the sodium bisulfite sequencing method harbors none of the two HhaI sites, two of the 11 HpaII, and 11 of the 54 CpG sites on a 1917 bp MseI fragment. Of 57 molecules sequenced from this region, 14 had no detectable CpG methylation (24.6%) that include unmethylated and HhaI-methylated input DNA since there are no HhaI sites within the PCR amplicon for sodium bisulfite sequencing (Table 2). Molecules methylated at HpaII sites and at all CpG sites account for 21.1% and 54.4% of the molecules sequenced from region 1, respectively (Table 2). Amplicon for region 2 contains both HpaII sites and 11 of the 12 CpG sites of the 283 bp MseI fragment, which has no HhaI sites, where it resides (Table 2). While 50% of the DNA from this 283 bp MseI fragment in the input has no CpG methylation and should not be pulled down by the antibody, 11.3% of the molecules sequenced from this region had no CpG methylation (Table 2). A total of 88.7% of the molecules sequenced was HpaII-methylated (4.8%) or fully methylated (Table 2). Region 3 amplicon includes two of the three HhaI sites, one of the three HpaII sites, 17 of the 23 CpG sites on a 1088 bp MseI fragment (Table 2). Among the molecules sequenced from this region, 10.3% was unmethylated, 4.4% was HhaI-methyalted, 8.8% was HpaII-methyalted, and 76.5% was fully methylated (Table 2). Although these sodium bisulfite sequencing assays are not considered quantitative, it is clear that unmethylated DNA is pulled down by the antibody at a level that should not be dismissed or neglected. It is noteworthy that DNA fragments with no methylation are detected in these assays at a frequency similar to or no lower than DNA fragments with two or three methylated sites. This finding is consistent with the results described above that anti-5-methyl-C antibody is not reliable in distinguish fully unmethylated DNA and DNA fragments with fewer than 10 methylated CpG sites.

**Table 2:** **Summary of number of molecules identified with different methylation patterns in IP assays using 5-methyl-C antibody with input DNA of mixed methylation patterns**

**Region**	**Methylation status**	**#CpG in**		**# of molecules detected in**		**% of total**
		**MseI frag.**	**Amplicon**	**Exp 1**	**Exp 2**	
1	Unmethylated	0	0	7	7	24.6
	Hha-methylated	2	0	0	0	
	HpaII-methylated	11	2	4	8	21.1
	Fully methylated	54	11	17	14	54.4
2	Unmethylated	0	0	3	4	11.3
	Hha-methylated	0	0	0	0	
	HpaII-methylated	2	1	0	3	4.8
	Fully methylated	12	11	27	25	83.9
3	unmethylated	0	0	2	5	10.3
	Hha-methylated	3	2	1	2	2.2
	HpaII-methylated	3	1	1	5	8.8
	Fully methylated	23	17	25	27	76.5

### Anti-MBD2 antibody pull down also increases with the number of CpG sites on the DNA fragment

**Figure 3:**
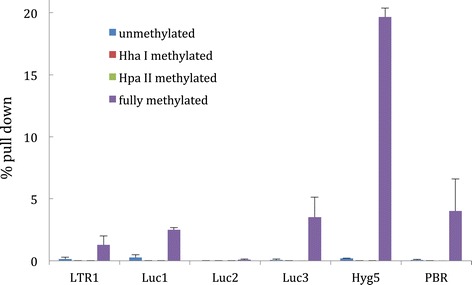
**Percent pull down by anti-MBD2 antibody increases with increasing number of methylated CpG sites on the target DNA fragment.** The Y-axis indicates the % pull down from IP assays of fully methylated target DNA.

Anti-MBD2 antibody is also commonly used to identify DNA with CpG methylation indirectly through the MBD2 protein. MseI restriction enzyme digested unmethylated, HhaI-methylated, HpaII-methylated, and fully methylated pCLH22 DNA was individually used as targets for IP with anti-MBD2 antibody and MBD2 protein to assess the feasibility of this assay. The percentage pull down of unmethylated DNA (Figure 3), much less than 0.5%, was clearly at the same level as the control assay without the antibody (data not shown) indicating a very low background of the IP using this antibody. However, the level of pull down of HhaI-methylated and the HpaII-methylated DNA fragments, which have 0 to 10 methyalted CpG sites, were also much less than 0.5% (Figure 3). The fully methylated DNA fragments were pulled down at a level from 0.1% for the fragments with seven methylated sites (i.e. Luc2) to nearly 20% for fragments with 143 methyalted sites (i.e. Hyg5) (Figure 3). These findings indicate that the capability of anti-MBD2 to pull down methylated DNA increases with the number of methylated CpG sites on the DNA fragment and the DNA can be reliably pulled down by the antibody when it has more than 20 methylated sites. It should be noted that we have no information on the feasibility or reliability for pulling down DNA with less than 21 and more than 10 methylated CpG sites.

### Specificity of anti-MBD2 may be affected by the nature of DNA fragment in addition to the number of methylation sites on the DNA

The same experiment of mixing differently methylated and unmethyalted target DNA in the IP followed by sodium bisulfite sequencing as described above using the anti-MBD2 and MBD2 protein was carried out to evaluate the specificity of the antibody. For regions 1 and 2, it appears that the antibody is much more specific for fully methylated DNA (93.9% and 82.4%, respectively) than unmethylated, HhaI-methylated, or HpaII-methylated DNA (Table 3). To our surprise, most of the molecules sequenced (80.4%) from region 3 were fully unmethylated (Table 3). As described above, all DNA regions examined by qPCR showed very low IP pull down of unmethylated targets. Since this DNA fragment was not assayed in the qPCR quantitative analysis, region 3 was amplified from sodium bisulfite treated DNA from two additional IP of mixed DNA target. Again, unmethylated DNA was preferentially pulled down by the antibody (Exp. 3 and Exp. 4 in Table 3). To ensure that this region can be amplified after sodium bisulfite treatment when the CpG methylation is present, the DNA pulled down from IP of MseI digested fully methylated pCLH 22 DNA was sequenced by the sodium bisulfite method. All 16 molecules sequenced from this control experiment in duplicate were fully methylated at CpG sites. It is unclear why anti-MBD2 antibody performs poorly in pulling down the 1088 bp MseI fragment within which the region 3 resides. The length of the DNA fragment does not appear to be an issue for this assay since region 1 assayed is on a DNA fragment of 1917 bp and region 2 is on a DNA fragment of 283 bp. Also, the number of methylated CpG sites does not seem to play a role in this phenomena since DNA fragment harbors region 2 has only 12 CpG sites. This 1088 bp fragment contains the EBV oriP region containing many repeat sequences, and this feature and the DNA sequence may cause the favorable binding of MBD2 protein and/or the antibody to this DNA fragment when it is unmethylated. The findings here indicate that while anti-MBD2 can pull down DNA fragments with more than 12 methylated CpG sites pretty reliably through MBD2 binding, it may be affected by DNA sequence or conformation.

**Table 3:** **Summary of number of molecules identified with different methylation patterns in IP assays using anti-MBD2 antibody with input DNA of mixed methylation patterns**

**Region**	**Methylation status**	**# CpG in**		**# of molecules detected**				**% of total**
		**MseI frag.**	**Ampl-icon**	**Exp 1**	**Exp 2**	**Exp 3**	**Exp 4**	
1	unmethylated	0	0	1	0			3.0
	Hha-methylated	2	0	0	0			
	HpaII-methylated	11	2	1	0			3.0
	Fully methylated	54	11	15	16			93.9
2	unmethylated	0	0	1	1			11.8
	Hha-methylated	0	0	0	0			
	HpaII-methylated	2	1	1	0			5.9
	Fully methylated	12	11	6	8			82.4
3	unmethylated	0	0	15	14	7	5	80.4
	Hha-methylated	3	2	0	0	0	0	0
	HpaII-methylated	3	1	0	0	0	0	0
	Fully methylated	23	17	0	6	1	3	19.6

## Conclusions

Both anti-5-methyl-C and anti-MBD2 antibodies can pull down DNA fragments with multiple methylated CpG sites. The capability of both of these antibodies to pull down methylated DNA increases with the number of CpG sites on the DNA fragment. Anti-5-methyl-C antibody can pull down unmethylated DNA at a non-negligible level around 2%, and it can pull down DNA fragments with more than 30 CpG sites reliably and consistently. While anti-MBD2 appears to have good specificity for pulling down highly methylated DNA with 12 or more CpG sites indirectly through the binding of MBD2 protein to the DNA, it may be affected by DNA sequence or conformation. Fully unmethylated DNA can be captured by both antibodies and create false positive findings. Also, some DNA fragments may bind to the beads used in the assay and create false positive finding. These types of false positive findings without rigorous confirmation had led to the report of 5-methyl-C presence in human mitochondrial DNA [5], which has been conclusively ruled out [6]. Also, DNA harboring less than 10 methylated sites can easily be missed by both antibodies and false negative findings can be derived. Parallel experiments using the two antibodies individually in the assay may provide one level of confirmation since the two antibodies may complement each other to reduce false positives and false negatives. In conclusion, both anti-5-methyl-C and anti-MBD2 antibodies can be used to identify DNA with multiple methylated CpG sites, preferably more than 10 sites, but great caution should be taken in interpreting the findings. In this study, we only tested each antibody from a single source, careful testing of antibodies from each and different vendors should be carried out to ensure the experimental results are correctly interpreted. Careful confirmation using sodium bisulfite sequencing method should be made before reaching a final conclusion.
